# Functional Superhydrophobic Surfaces with Spatially Programmable Adhesion

**DOI:** 10.3390/polym12122968

**Published:** 2020-12-12

**Authors:** Duan-Yi Guo, Cheng-Huan Li, Li-Min Chang, Hung-Chang Jau, Wei-Chun Lo, Wei-Chun Lin, Chun-Ta Wang, Tsung-Hsien Lin

**Affiliations:** Department of Photonics, National Sun Yat-sen University, Kaohsiung 80424, Taiwan; alex20150131@gmail.com (D.-Y.G.); alex082668@gmail.com (C.-H.L.); book74108520@gmail.com (L.-M.C.); hcjau@staff.nsysu.edu.tw (H.-C.J.); d0355236@itd.fcu.edu.tw (W.-C.L.); wclin@mail.nsysu.edu.tw (W.-C.L.); wangchunta4@gmail.com (C.-T.W.)

**Keywords:** superhydrophobic surfaces, lotus effect, petal effect, liquid crystal, photopolymerizations

## Abstract

A superhydrophobic surface that has controllable adhesion and is characterized by the lotus and petal effects is a powerful tool for the manipulation of liquid droplets. Such a surface has considerable potential in many domains, such as biomedicine, enhanced Raman scattering, and smart surfaces. There have been many attempts to fabricate superhydrophobic films; however, most of the fabricated films had uniform adhesion over their area. A patterned superhydrophobic surface with spatially controllable adhesion allows for increased functions in the context of droplet manipulation. In this study, we proposed a method based on liquid-crystal/polymer phase separation and local photopolymerization to realize a superhydrophobic surface with spatially varying adhesion. Materials and topographic structures were analyzed to understand their adhesion mechanisms. Two patterned surfaces with varying adhesion were fabricated from a superhydrophobic material to function as droplet guides and droplet collectors. Due to their easy fabrication and high functionality, superhydrophobic surfaces have high potential for being used in the fabrication of smart liquid-droplet-controlling surfaces for practical applications.

## 1. Introduction

A superhydrophobic surface has potential for manipulating the flow of liquid droplets. The high contact angle of a superhydrophobic surface enables liquids to exist in the droplet form on it. A superhydrophobic surface allows for separated droplet manipulation to minimize the crosstalk among droplets and transportation loss from it. Due to their non-wettability, superhydrophobic surfaces have been applied in various domains, such as drug discovery [1,2], bioassays [3,4], enhanced Raman scattering [5,6], and the characterization of ice formation [7,8]. According to the level of the adhesion force, the superhydrophobicity effect is categorized into the lotus effect (weak adhesion force) and the petal effect (strong adhesion force) [9,10,11,12]. A lotus-effect surface is characterized by a high water-contact angle and a low sliding angle for droplet rolling [13]. Lotus-effect surfaces are named so because they mimic the surfaces of lotus leaves. Due to the weak adhesion of a lotus-effect surface, water droplets easily roll off it and carry particles (e.g., dust) with them [14]. Due to their weak adhesion, lotus-effect surfaces have been applied in many domains, such as self-cleaning [15,16,17,18], drag reduction [19,20,21], and water harvesting [22,23]. In contrast to the droplet-repelling characteristic of lotus-effect ones, petal-effect surfaces have a strong adhesion force that pins water droplets to their surface but at high water-contact angles [11,12,24]. By spatially adjusting the adhesion force on a superhydrophobic surface, a water droplet can be feasibly directed to preprogrammed positions (e.g., in lab-on-a-paper devices [25,26]) for research or practical purposes. Therefore, a patterned superhydrophobic surface with spatially controllable adhesion is expected to act as a smart superhydrophobic surface. Such surfaces are versatile and capable of performing various functions related to the control of water droplets.

According to a large body of experimental findings [9,27,28,29,30,31] and theoretical analyses [13,32,33], the aforementioned two effects occur due to topographical morphologies and chemical properties of surface constituents. A superhydrophobic surface must be rough with micro- and nano-sized hierarchical structures and must be constituted of hydrophobic materials. The Cassie–Baxter and Wenzel wetting equations are well-known models that describe the contact angle on a rough surface [34]. The Cassie–Baxter model, which is based on the Cassie wetting model, describes the high contact angle of a heterogeneous wetting condition by introducing the concept of an air pocket. The Cassie–Baxter model is expressed in Equation (1).

(1) $$\;cos\theta =r\cdot f\cdot cos\theta_{y}+f-1$$

where *θ* is the contact angle, *r* is the roughness factor, *f* is the fraction of the solid surface that is wet, *θ_y_* is Young’s contact angle on the flat solid surface, and 1 − *f* is the fraction of the area occupied by an air pocket. Because of the high contact angle (approximately 180°) at the air–liquid interface, air pockets increase the contact angle and contribute to the surface’s easy-rolling characteristic (i.e., the lotus effect). In the case of a totally wetted surface (i.e., *f* = 1), the wetting state is subjected to Wenzel wetting regime, which is governed by Equation (2).

(2) $$cos\theta =r\cdot cos\theta_{y}\;$$

In the aforementioned regime, the contact angle is governed by the roughness of a solid surface. A high roughness factor can result in a high contact angle for a hydrophobic surface (*θ_y_* > 90°). Moreover, a water droplet is more liable to be pinned by the hierarchical structure of a rough surface owing to the behavior of the surface in the total wetting state. Accordingly, air pockets are crucial for inducing lotus effect or petal effect, and the formation of air pockets is determined by the surface topography. Many attempts have been made to realize lotus-effect and petal-effect surfaces, usually by adjusting the aspect ratio of a surface pattern or controlling the size of hollow holes in a porous structure. Electrospinning [35,36,37], plasma etching [38,39], solution immersion [40,41], laser writing [42,43,44], electrodeposition [28,45], and self-assembly process [46,47,48] are commonly employed to fabricate these two types of hydrophobic surfaces. Moreover, the multi-fluorination strategy is used to enhance the robustness of a superhydrophobic surface made by self-stratifying coating [49,50]. Photoresponsive surfaces based on photo-isomerization have been reported on realizing a patterned superhydrophobic surface with spatially and dynamically controllable adhesion [36,37,51,52,53]. Nonetheless, most surfaces fabricated by the aforementioned methods exhibit the lotus or petal effect but not both effects. In addition, the realization of a stable superhydrophobic surface with spatially controllable adhesion is demanding and requires a different method.

Herein, we formulate a system featuring liquid-crystal/polymers phase separation in combination with spatial exposure and the washing out technique. This system is used to fabricate a porous polymer structure to realize a superhydrophobic surface with spatially controllable adhesion. The phase separation process can be used to easily configure the surface’s hierarchy according to the temperature-dependent mutual solubility of monomers and liquid crystals. In addition, two polymer materials with different chemical properties are employed to adjust the surface chemistry of the polymer films in the phase separation process. In this proof-of-concept work, a superhydrophobic surface with controllable adhesion is accomplished by the proposed method, instead of a conventional fabrication process. Scanning electron microscope (SEM) and atomic force microscope (AFM) images are presented to detail and explain the surface’s wetting characteristics. X-ray photoelectron spectroscopy (XPS) is used to confirm the surface chemistry of the polymer films with different fabrication temperatures. In addition, two patterned superhydrophobic surfaces are demonstrated to act as functional superhydrophobic surfaces.

## 2. Materials and Methods

### 2.1. Sample Fabrication

**Figure 1:**
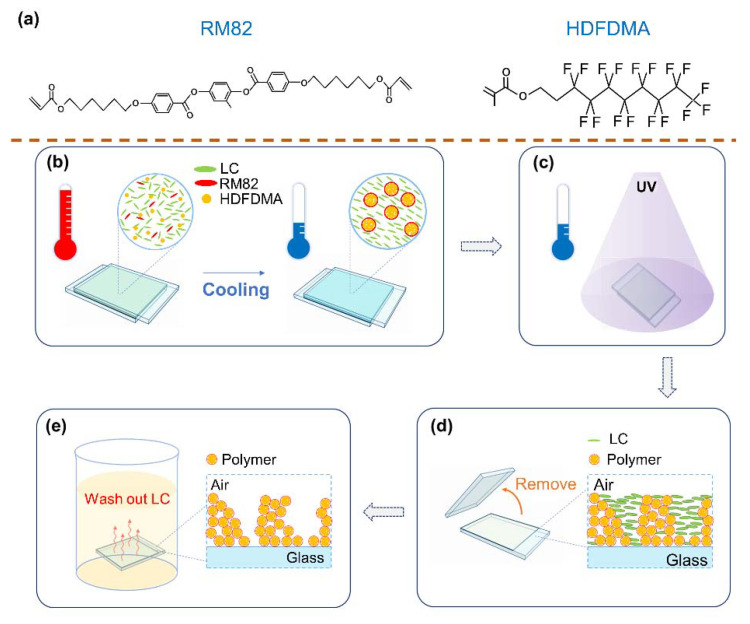
Scheme of the fabrication process. (**a**) Chemical structures of diacrylate monomer (RM82) and hyptadecafluorodecyl methacrylate (HDFDMA). (**b**) Phase separation by direct cooling. (**c**) Photopolymerization by ultraviolet (UV) light. (**d**) Removal of the upper substrate. Inset scheme of the sample after the substrate removal. (**e**) Washing out unpolymerized (liquid crystal) LC. Inset: Scheme of the sample with LC washed out.

Two mixtures (denoted by Mixture 1 and 2) were prepared for the fabrication of a superhydrophobic surface. Mixture 1 was composed of the following materials: HTW114200-050 [nematic liquid crystal (NLC); nematic phase lies between −40 °C and 107 °C; from HCCH, Yangzhong, China], hyptadecafluorodecyl methacrylate (HDFDMA, from Sigma-Aldrich, Taufkirchen, Germany), trimethylolpropane triacrylate (TMPTA, from Alfa Aesar, Ward Hill, MA, US), and irgacure 651 (IRG651, photo-initiator). Mixture 2 was composed of the following materials: HTW114200-050, HDFDMA, TMPTA, diacrylate monomer RM82 (from HCCH, Yangzhong, China), and IRG651. The chemical structures of RM82 and HDFDMA are shown in Figure 1a. The role that NLC plays in the fabrication is to define the configuration of polymer fibrils through phase separation and to create a porous structure after being washed out from the polymer structure. HDFDMA is a hydrophobic molecule because of the trifluoromethyl group (–CF3) and the fluorine-contained carbon chain. Notably, RM82 is more hydrophilic than HDFDMA due to RM82′s polar functional group. Thus, RM82 is used to adjust the chemical properties of the polymer template. The mixtures were heated to an isotropic state and then injected into a cell that comprised two glasses with a 3-μm gap between them. The surfaces of the substrates were coated with homogeneous alignment polyimide SE-5291 (from Nissan, Tokyo, Japan) and polyvinyl alcohol (PVA), respectively. Figure 1 illustrates the scheme underlying the fabrication process. Initially, the sample is heated to 110 °C (the isotropic state) and then cooled to a certain temperature (60 °C and 30 °C in this study) at 1 °C/min (Figure 1b). In this step, the aim is to control the configuration of the mixture through the thermally induced phase separation process. After the cooling process, the sample is illuminated with ultraviolet (UV) light (XLite380, from OPAS, Taichung city, Taiwan; centered at approximately 365 nm) with an intensity of 30 mW/cm^2^ to undergo polymerization (Figure 1c). As displayed in Figure 1d, the top substrate of the sample is removed to have an open surface. The inset in Figure 1d is the schematic of the polymer structure with unpolymerized mesogens. Finally, the sample is immersed in hexane to wash out the unpolymerized mesogens to obtain a porous polymer structure (Figure 1e).

### 2.2. Characterization

Polarized microscope images of the polymer structure captured using a polarized optical microscope (Eclipse LV 100 POL, from Nikon, Melville, NY, US), and higher-resolution images were captured using a scanning electron microscope (Phenom ProX, from KE CHIEH Tech, New Taipei City, Taiwan). In addition, an atomic force microscope (E-sweep system, from Seiko, Chiba, Japan) was used to image the topographic configuration. To measure the water-contact angle on the superhydrophobic surface, we used a drop shape analyzer (DSA-100, from KRÜSS Optronic, Hamburg, Germany). Notice that what we measured was a static water contact angle. The surface chemical analysis was performed with a PHI 5000 VersaProbe (Chigasaki, Japan) X-ray photoelectron spectroscopy (XPS) system. Microfocused Al Kα X-rays were used, and the take-off angle of the photoelectron beam was 45°. The X-ray and acceptance lens of the analyzer was rastered over an area of 500 μm × 500 μm. The detection limit in depth of the XPS system is ~6 nm.

## 3. Results and Discussion

### 3.1. Investigation of Polymer Constituents

**Figure 2:**
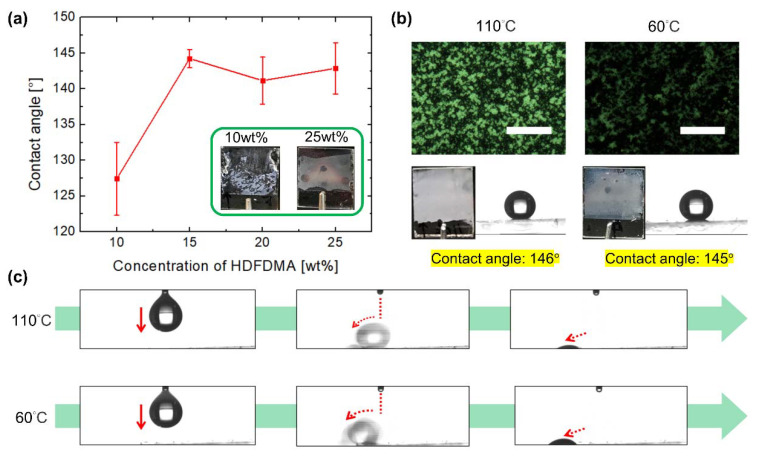
Water wetting on a superhydrophobic surface fabricated from mixture 1. (**a**) Contact angles of a surfaces fabricated from Mixture 1, which was composed of ratio-varying LC and HDFDMA. Inset: photos of polymer films of 10 and 25 wt% HDFDMA on the left and right, respectively. (**b**) Polarized optical microscope images, photographs, and contact angles of the surfaces fabricated at 110 and 60 °C. Scale bars: 50 μm. (**c**) From left to right, frames of water droplets flowing off the surfaces.

Figure 2 displays the wetting characteristics of a single water droplet on the superhydrophobic surfaces fabricated from Mixture 1. To examine the robustness and superhydrophobicity of a polymer film, the weight ratio of HDFDMA was first examined through the polymer film fabrication, as illustrated in Figure 1a. The weight ratio of HDFDMA increased from 10.0 to 15.0, 20.0, and 25.0 wt%, and those of NLC decreased from 87.5 to 82.5, 77.5, and 72.5 wt% when using 2.0 wt% TMPTA and 0.5 wt% IRG651, respectively. All samples were rapidly cooled from the isotropic state to room temperature (approximately 25 °C) and then photopolymerized with UV light. As the weight ratio of HDFDMA increased, the contact angle of the water droplet on the films increased and the polymer fibrils became more robust against damage from the fabrication process (e.g., when LC was washed out), as illustrated in the insets of Figure 2a. The result suggests that high-weight-ratio HDFDMA is essential for fabricating a robust and superhydrophobic film. However, the excessive concentration of polymer may decrease the contact angle because of the presence of an insufficiently rough dense polymer fibril, simultaneously reducing the tunability contributed by NLC. Moreover, an all-polymer film that contains no NLC has a practically flat surface such that the water-contact angle is only approximately 127° ± 5.1°, which represents a normal hydrophobic state (data not shown). NLC is crucial for producing a porous structure to obtain a rough surface. Therefore, we use 20–25 wt% HDFDMA to fabricate a superhydrophobic film. Furthermore, the films were fabricated at 110 and 60 °C, respectively, to investigate the influence of the topographical differences on the water wetting behavior. After the surfaces were fabricated (see the sample fabrication subsection), polarized optical microscope (POM) images indicated the presence of porous-like polymer structures with hollow holes of different sizes ranging from a few microns to a few tens of microns (Figure 2b). Moreover, as indicated in the photos, the presence of opaque films due to Mie scattering suggested that the domain size of the porous structure was of the order of a few microns to tens of microns. Because of the micrometer-sized polymer structure and fluorine-containing functional group of constituents, the water-contact angles of the surfaces reach 146° and 145°, respectively, which indicated that both were superhydrophobic and could make water in the form of droplets. As shown in Figure 2c, a 10 μL water droplet was repelled by both surfaces and rolled off upon contact with the surfaces, which suggested that the lotus effect, rather than the petal effect, was dominant in the aforementioned surfaces (see Supplementary Video S1).

**Figure 3:**
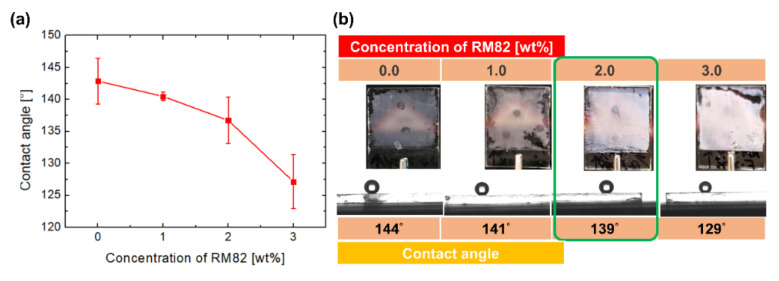
Water wetting on the superhydrophobic surface fabricated from Mixture 2. (**a**) Relationship between the concentration of RM82 and the contact angle. (**b**) Series photos and contact angles of surfaces made from RM82 of different concentration.

To adjust the adhesion of the superhydrophobic film, RM82 was introduced into Mixture 2. The weight ratio of RM82 in Mixture 2 varies from 0 to 0.1, 1.0, 2.0, and 3.0 wt% when 25.0 wt% HDFDMA, 2.0 wt% TMPTA, and 0.5 wt% IRG651 (the remainder was NLC) were doped into the mixture. All the samples were rapidly cooled from the isotropic state and fabricated at room temperature. As depicted in Figure 3a, the contact angle changes from 142.9° ± 3.6° to 127.2° ± 4.2° as the weight ratio of RM82 increased. Furthermore, the downward trend of the contact angle indicated that the surface tended to be hydrophobic. This result suggested that at increasing amounts, RM82 increasingly dominates the wettability of a surface due to its polar functional group, which cause the surface to experience a normal hydrophobic regime. Besides, the photos showing that the films become opaquer indicate the higher density of the polymer films (Figure 3b). As mentioned in the last paragraph, a dense polymer film may lose a porous structure and bring about a flat surface. Therefore, we selected a 2.0 wt% RM82-containing mixture. This mixture enhanced the tunability of film fabrication by enabling surface adhesion control while maintaining a high contact angle. Thus, by adopting the aforementioned mixture, we fabricated an adhesion-controllable superhydrophobic surface.

### 3.2. Lotus or Petal Effect of the Surface

**Figure 4:**
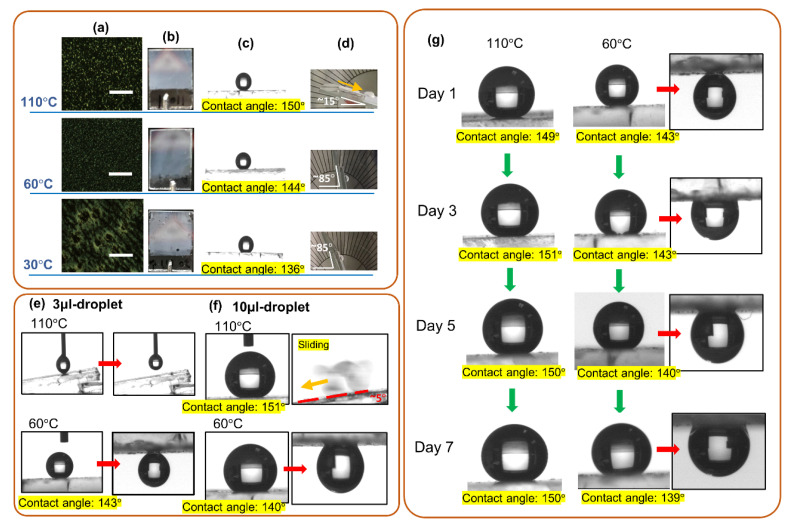
(**a**) Polarized optical microscope (POM) images, (**b**) photographs, (**c**) water-contact angles, (**d**) water sliding angles of the sample fabricated at 110, 60, and 30 °C. Scale bars in (**a**) are 50 μm. (**e**) Water-contact angles and sliding angles of the sample fabricated at 110 and 60 °C by using a 3 μL water droplet. (**f**) Water-contact angles and sliding angles of the sample fabricated at 110 and 60 °C by using a 10 μL water droplet. (**g**) The long-term stability of the sample fabricated at 110 and 60 °C, respectively.

Considering the results presented in the last two paragraphs, we used the mixture composed of 75.5 wt% HTW114200-050, 20.0 wt% HDFDMA, 2.0 wt% TMPTA, 2.0 wt% RM82, and 0.5 wt% IRG651 as a precursor to fabricate an adhesion-controllable superhydrophobic surface. Thermally induced phase separation (TIPS) and polymerization-induced phase separation (PIPS) were employed to control the configuration of polymer network, which is a crucial element in directing the surface toward water-repellency or water-pinning ones. The POM images in Figure 4a indicate the presence of a micro-sized porous polymer structure with sizes ranging from a few microns to tens of microns. Figure 4b indicates that all the polymer films were opaque due to Mie scattering. According to the POM images, among the three polymer films, the polymer film fabricated at 60 °C exhibited the densest dots (indicating the position polymer occupying) and highest area fraction of the edge. As shown in Figure 4c,d, the water-contact angles of the three surfaces decreased (from 150° to 144° and 139°) with the fabricated temperatures, whereas their water sliding angles increased with the fabricated temperatures (see Supplementary Video S2). According to the semi-empirical Furmidge equation [54], the adhesive force (*F_ad_*) of a surface can be derived from the relation among the receding contact angle (*θ_R_*), the advancing angle (*θ_A_*), the water surface tension (*γ_water_*), and the width of the water-solid interface (*W_dro_*), which is ruled by Equation (3).

(3) $$\frac{F_{ad}}{W_{dro}}=\gamma_{water}\cdot \left(cos\theta_{R}-cos\theta_{A}\right)$$

The *θ_R_* and *θ_A_* of the sample fabricated at 110 °C were 144.9° ± 0.5° and 151.3° ± 2.6°, respectively; whereas the *θ_R_* and *θ_A_* of the sample fabricated at 60 °C were 75.9° ± 8.8° and 137.9° ± 5.5°. Thus, the adhesive forces of the sample fabricated at 110 and 60 °C were 5.7 ± 2.7 μN and 104.9 ± 9.3 μN. These results suggested that the film fabricated at 110 °C was a water-repellency and had a surface where the lotus effect dominated; whereas the film fabricated at 60 and 30 °C had a water droplet pinned onto its surface, where the petal effect dominated. As shown in Figure 4e,f, the superhydrophobicity of the surfaces was examined by water droplets with different volumes (3 μL and 10 μL were used in this experiment). Figure 4e shows the behaviors of 3 μL-droplets on a film fabricated at 110 and 60 °C, respectively. For the film fabricated at 110 °C, the surface was water-repelling so that a water droplet could not be attached to the surface. Notice that the water droplet was too small to fall from the tube spontaneously (see Supplementary Video S3). However, for the film fabricated at 60 °C, a water droplet was stuck at the surface with the contact angle of 143°, showing that it was a petal-effect-dominated surface. In the case of a water droplet with a volume of 10 μL, as shown in Figure 4f, both samples were still superhydrophobic. For the film fabricated at 110 °C, the contact angle was 151° and the sliding angle was 5° (see Supplementary Video S3). For the film fabricated at 60 °C, the contact angle was 140° and a water droplet was still stuck at the upside-down surface. Thus, the results indicated the two films were available for manipulating a water droplet with a volume ranging from 3 μL to 10 μL. In addition, the stability of the films was checked, as shown in Figure 4g. The film fabricated at 110 °C remained a high contact angle (150°) and low sliding angle (5°) after a week (see Supplementary Video S4). The contact angle of the film fabricated at 60 °C was ~139° with a neglectable decrease (~4°), and a water droplet could be stuck at the upside-down surface after a week.

**Figure 5:**
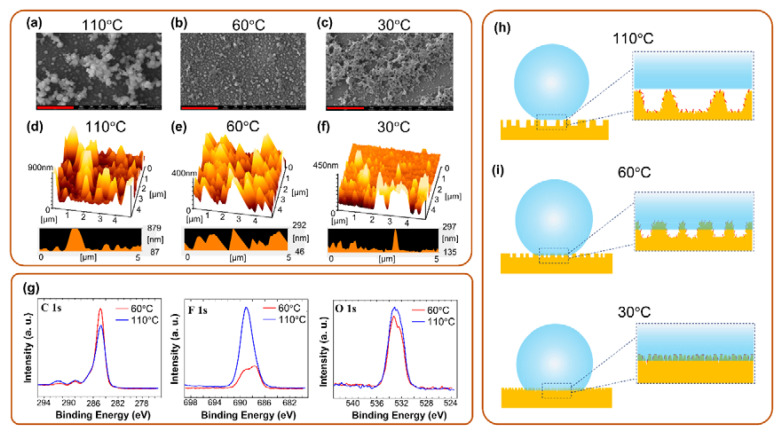
(**a**–**c**) Scanning electron microscope (SEM) and (**d–f**) atomic force microscope (AFM) images of the samples fabricated at 110, 60, and 30 °C. Scale bars in (**a**–**c**) are 5 μm. (**g**) The XPS C1s, F1s, and O1s spectra of sample fabricated at 110 and 60 °C. (**h**) Scheme of the samples fabricated at 110 °C. (**i**) Scheme of the samples fabricated at 60, and 30 °C.

The surfaces of the polymer films were also characterized through AFM and SEM to determine the surface morphologies at the nanometer scale. The surface chemistry of the polymer films were examined by XPS system to confirm the chemical state and the surface composition. As shown in Figure 5a, the polymer fabricated at 110 °C had a sphere-like morphology with its constituents piled up in heaps. According to Figure 5d, the distance between heaps was approximately 5 μm, and the height of a heap could approximately 800 nm, which was the highest value among the three films (Figure 5e,f). Such high heaps prevented a water droplet from directly contacting the surface and accordingly made air pockets form. Compared with the one fabricated at 110 °C, the polymer fabricated at 60 °C also had a sphere-like morphology but its constituents were distributed uniformly with a domain size of approximately 1 μm (Figure 5b). The topographic image (Figure 5e) indicates that the depth difference of the rough surface was approximately 200nm and that the spacing between the peaks was <1 μm. The relatively flat surface potentially allowed itself to be immersed completely by a water droplet; thus, the petal effect dominated on this surface. Both the aforementioned films had micrometer-sized rough surfaces and consequently high contact angles. For the polymer fabricated at 30 °C had a sponge-like topography with a reduced depth difference and practically flat surface (Figure 5c,f). This polymer also exhibited the petal effect but at a lower contact angle than the polymers fabricated at 110 and 60 °C did. In Figure 5g, the XPS F1s spectrum illustrates a stronger fluorine signal on the film surface fabricated at 110 °C. The C1s spectrum also confirms a higher amount of C-F bonding (~292 eV) on the surface of film fabricated at 110 °C. Table 1 lists the surface composition of films fabricated at 110 and 60 °C, respectively. It is noticed that the fluorine on the sample fabricated at 60 °C decreases significantly by 9% compared to the one fabricated at 110 °C. This composition difference may also attribute to the wettability of the film surface. Based on these results, the scheme of the mechanism is illustrated in Figure 5h,i. Different mutual solubility and diffusion rates of materials (NLC, RM82, and HDFDMA) in the two-phase separation process (TIPS followed by PIPS) play an important role in controlling the surface topography and chemistry. Due to the high diffusion rate at 110 °C, the monomers and NLC were effectively separated in the PIPS process. Thus, monomers were more easily merged to form a rough surface and large droplet-like polymer domain. Besides, RM82 was dissolved well with HDFDMA at the high temperature, so the effect exerted by the RM82 on the wettability was buried with HDFDMA. Thus, as illustrated in Figure 5h, for the film fabricated at 110 °C, a rough surface due to a porous polymer film resulted in a high contact angle and a surface where the lotus effect dominated due to the presence of air pockets. Because of the reduced solubility at the lower temperature, 60 and 30 °C, the materials were initially separated in the cooling process prior to exposure to UV light, and the degree of diffusion in the PIPS process was lower than the one at 110 °C. According to the fabrication parameters, the domain size of the porous structure was smaller than the one at 110 °C. Moreover, RM82 might separate from HDFDMA so that it exerted a non-negligible effect in increasing the surface adhesion of the polymer structure. Therefore, the film fabricated at 60 and 30 °C pinned a water droplet instead of repelling it. As schematics in Figure 5i, the reduced depth of the rough surface and the influence of RM82 made it easy for a water droplet to be pinned on the surface. Thus, the petal effect dominated on this surface.

### 3.3. Demonstration

**Figure 6:**
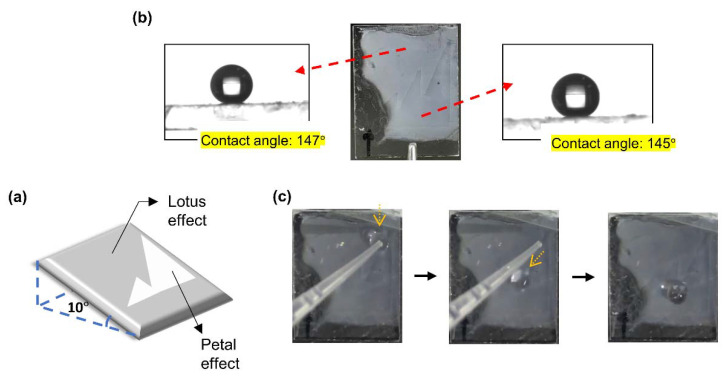
Demonstration of directional water droplet transport. (**a**) Scheme of the demonstration. (**b**) Photograph of the patterned surface and water contact angles of the two regions. (**c**) Frames of water droplet flow along the patterned line.

**Figure 7:**
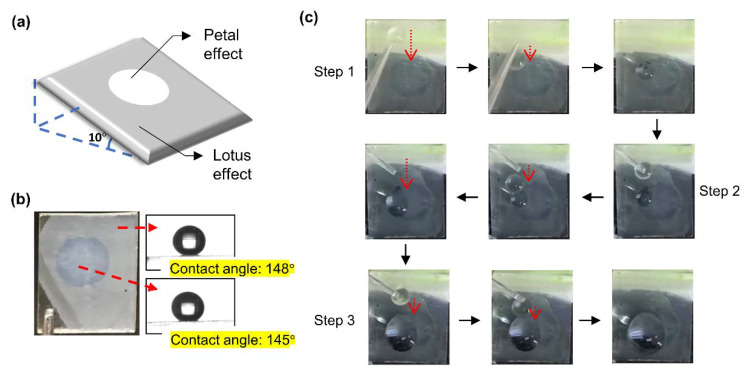
Demonstration of water droplet collection. (**a**) Scheme of the demonstration. (**b**) Photograph of the patterned surface and water contact angles of the two regions. (**c**) Frames of water-droplet collection.

To realize a functional superhydrophobic surface where a liquid droplet can be manipulated, we fabricated a superhydrophobic surface with spatially varying adhesion. The sample was first heated up to 110 °C and then spatially photopolymerized by introducing a mask whose function was to design a pattern. After the first step of polymerization, the sample was cooled to 60 °C, and the unpolymerized area was illuminated with UV light to conduct polymerization as per the method described in the sample fabrication subsection. Thus, the surface was divided into two areas in which the lotus effect or petal effect dominated. The efficacies of the two functional superhydrophobic surfaces were demonstrated in the context of water-droplet transportation and collection (Figure 6 and Figure 7 for each surface, respectively). Figure 6a illustrates the scheme underlying the superhydrophobic surface, which functioned as a water-droplet transporter. We designed a blaze-like pattern in one region to induce a petal effect, and the other region exhibited a lotus effect. The contact angles of the two surfaces were 147° and 145°, which indicated superhydrophobicity (Figure 6b). The sample was tilted by 10° to demonstrate a water droplet flowing and pinning. As displayed in Figure 6c, a water droplet dripped down the lotus-effect surface in the direction of gravitational pull. Upon coming into contact with the interface joining the two surfaces, the droplet flowed along the slant line until advancing into the petal-effect surface before being stopped (see Supplementary Video S5). Figure 7a illustrates the scheme underlying the superhydrophobic surface that functioned as a water-droplet collector. The petal effect was dominant in a small circular area, outside which the lotus effect dominated. The contact angle was high in both areas (Figure 7b). The sample was also tilted by 10° for the demonstration. As shown in Figure 7c, a water droplet dripped from the lotus-effect surface into the circular area before being trapped. After the three droplets dripped into this circular area, the three droplets were collected at the petal-effect area (see Supplementary Video S6).

## 4. Conclusions

In this work, we successfully fabricated a superhydrophobic surface with spatially controllable adhesion and demonstrated the efficacy of functional superhydrophobic surfaces. Owing to its partial order and fluidity, NLC is essential for controlling the configuration of polymers in the two-phase phase separation process. With suitable control in the cooling and photopolymerization process, the surface of the polymer film can exhibit lotus effect, petal effect, and both effects simultaneously. Moreover, XPS measurement and the nanometer-scale images captured through SEM and AFM indicated that the fabricated surface had a hierarchical structure that enabled two wetting states to occur. In the proof-of-concept study, we demonstrated that the NLC-polymer system can be feasibly applied to the field of a superhydrophobic surface. Considering the ease of organic and inorganic coating, we expect our polymer film to be further functionalized for various applications with its adhesion-controllable superhydrophobicity. Moreover, with the various assembly of nematic liquid crystal, it is anticipated that polymer structures possess intriguing optical properties, such as structural colors and optical resonance. Thus, the proposed method will also provide a promising avenue for integrated optical/superhydrophobic applications.

## Figures and Tables

**Table 1:** The surface composition of films fabricated at 110 and 60 °C.

Fabricated Temperature/Element	C 1s	O 1s	F 1s	In 3d5	Sn 3d
110 °C	84.11%	10.39%	5.49%	<0.1%	<0.1%
60 °C	73.69%	11.72%	14.54%	<0.1%	<0.1%

## Supplementary Materials

The following are available online at https://www.mdpi.com/2073-4360/12/12/2968/s1, Video S1: A water droplet flowing off the surface fabricated from Mixture 1 and at 110 °C and 60 °C. Video S2: The sliding angle measurement of the surface fabricated from Mixture 2 and at 110 °C, 60 °C, and 30 °C. Video S3: The sliding angle measurement of the surface fabricated from Mixture 2 and at 110 °C by using 3 μL- and 10 μL-water droplet. Video S4: The long-term stability of the sample fabricated at 110 °C. Video S5: Demonstration of directional water droplet transport. Video S6: Demonstration of water droplet collection.
